# Reduction of c-Fos via Overexpression of miR-34a Results in Enhancement of TNF- Production by LPS in Neutrophils from Myelodysplastic Syndrome Patients

**DOI:** 10.1371/journal.pone.0158527

**Published:** 2016-08-11

**Authors:** Yayoi Shikama, Meiwan Cao, Tomoyuki Ono, Xiaomin Feng, Hideyoshi Noji, Hideo Kimura, Kazuei Ogawa, Yuko Suzuki, Kazuhiko Ikeda, Yasuchika Takeishi, Junko Kimura

**Affiliations:** 1 Department of Pharmacology, Fukushima Medical University, Fukushima, Japan; 2 Department of Cardiology and Hematology, Fukushima Medical University, Fukushima, Japan; 3 Department of Hematology, Kita Fukushima Medical Center, Date, Japan; 4 Department of Blood Transfusion and Transplantation Immunology, Fukushima Medical University, Fukushima, Japan; University of Massachusetts Medical School, UNITED STATES

## Abstract

Although increased TNF-**α** has been considered to cause ineffective hematopoiesis in myelodysplastic syndromes (MDS), the mechanisms of TNF-**α** elevation are not known. We recently found that c-Fos mRNA stabilization under translation-inhibiting stimuli was impaired in MDS-derived neutrophilic granulocytes. In the current study, we identified overexpression of c-Fos-targeting miR-34a and miR-155 as the cause of impairment. Expression levels of miR-34a but not miR-155 inversely correlated with ratios of c-Fos-positive cells in MDS-derived CD16^+^ neutrophils (r = -0.618, *P*<0.05), which were analyzed by flow cytometry. Among the seventeen patients, c-Fos was detectable in less than 60% of CD16^+^ cells in eight patients (Group A), while five (Group B) expressed c-Fos in more than 80% of CD16^+^ cells, which was consistent with the controls (88.6 ± 7.8%). Group A-derived granulocytes secreted more TNF-**α** in response to 1 μM LPS for 3 hours (735.4 ± 237.5 pg/mL) than Group B (143.5 ± 65.7 pg/mL, *P*<0.05) and healthy controls (150.8 ± 91.5 pg/mL, *P*<0.05). Knockdown of c-Fos in neutrophil-like differentiated HL60 increased the binding of NF-κB p65 to the promoter region of TNF-**α** DNA. Thus, c-Fos reduction via overexpression of miR-34a contributes to TNF-**α** overproduction under inflammatory stimuli in MDS.

## Introduction

Myelodysplastic syndromes (MDS) are a heterogeneous group of hematological disorders characterized by ineffective hematopoiesis that results in refractory cytopenia with morphological and functional abnormalities, as well as higher susceptibility to leukemia [1–4]. However, the molecular basis of ineffective hematopoiesis has yet to be clarified. Peripheral blood cytopenia accompanied by hypercellularity in bone marrow (BM) has been considered to result from increased apoptosis of hematopoietic progenitors [5]. Upregulation of tumor necrotizing factor (TNF)- **α**, a proapoptotic cytokine, has been commonly observed in BM plasma and peripheral mononuclear cells, and is positively correlated with the degree of apoptosis in early stage/low-risk MDS [5]. TNF-**α** is a potent stimulator of nuclear factor kappa-light-chain-enhancer of activated B cells (NF-κB) that is increasingly recognized as playing a crucial role in malignancy development [6–9]. Moreover, TNF-**α** itself is also transcribed by NF-κB. Therefore, upregulation of TNF-**α** could play key roles in both the development of ineffective hematopoiesis and the progression of MDS.

One of the components of transcription factor activator protein 1 (AP-1) [10], c-Fos, regulates apoptosis and production of inflammatory mediators in neutrophils [11–14], and was recently shown to act as a suppressor of LPS-induced production of cytokines including TNF-**α** via physical interaction with p65 protein of NF-κB [15]. The c-Fos transcription factor is encoded by *FOS*, an immediate early gene whose transcripts have a short life span and rapidly increase in response to a wide range of stimuli including the cellular stress that induces cessation of cap-dependent translation [16–19]. The rapid increase of *FOS* mRNA is attributable to transcriptional regulation [19, 20] and post-transcriptional regulation, which decelerates *FOS* mRNA decay [21, 22]. We recently found that the elevation of c-Fos mRNA following translation arrest was attenuated because of insufficient mRNA stabilization in granulocytes isolated from MDS patients [23, 24]. Since deregulated expression of c-Fos could affect TNF-**α** production, the aim of the present study was to clarify the causes and consequences of the impaired stabilization of *FOS* mRNA in MDS.

The life span of *FOS* mRNA is regulated via its 3’UTR [21], which includes target sequences for multiple microRNAs (miRNAs) [25–28] and an AU-rich element (ARE) [29, 30] where Hu antigen R (HuR) [31–33], an mRNA-stabilizing protein, and AUF1, which generally destabilizes its target mRNA [34, 35]. Although *FOS* mRNA stabilization following translation arrest was accompanied by the association of HuR with an ARE in *FOS* mRNA, our previous study showed that the majority of MDS patients expressed similar levels of HuR protein in granulocytes compared to the healthy controls. No involvement of AUF1 in *FOS* mRNA stabilization under translation arrest was detected, and no mutations were found in *FOS* mRNA 3’UTR [24]. Therefore, miRNAs were likely to be the cause of the impaired stabilization of *FOS* mRNA observed in MDS.

It has been shown that miRNAs play crucial roles in hematopoiesis. For example, the maturation of BM-derived dendritic cells requires silencing of c-Fos by miR-155 in both human and mice [25]. Additionally, miRNAs are involved in the development of malignancies, and aberrant miRNA expressions have been reported in various malignant diseases, such as the elevation of miR-155 and miR-125b in acute myelogenous leukemia [25, 36] and the decrease of miR-34a in various solid organ cancers [37–39]. In MDS, miR-378, miR-632, and miR-636 were increased in BM-derived mononuclear cells [40]. CD34^+^ cells isolated from patients classified into subtypes with a high risk of leukemic transformation overexpressed miR-155 and miR-210 [41]. In low-risk MDS subtypes, a decrease of let-7a and miR-16 in plasma [42] and an increase of miR-34a in CD 34^+^ cells [43] have been reported. Thus, abnormal miRNA expression possibly affects *FOS* mRNA stabilization under translation arrest in MDS-derived neutrophils.

In this study, we identified *FOS*-targeting miRNAs that were overexpressed in granulocytes from patients with early-stage MDS and their effects on expression of c-Fos protein. We also demonstrated that the reduction of c-Fos led to excessive production of TNF-**α** in response to LPS.

## Materials and Methods

### Patients and granulocyte isolation

Peripheral blood was obtained from 23 patients with MDS consisting, according to the 2008 WHO classification, of four cases of refractory cytopenia with unilineage dysplasia (RCUD), seventeen cases of refractory cytopenia with multilineage dysplasia (RCMD), two cases of refractory anemia with excess blasts-1 (RABE-1), and 17 age-matched healthy controls. All donors provided written informed consent in accordance with the Institutional Human Research Committee and Helsinki Declaration [44] developed by the World Medical Association. The neutrophil fraction was obtained by centrifugation through Lymphoprep (l.077 g/mL, Axis-Shield, Oslo, Norway) followed by hypotonic lysis of erythrocytes, as previously described [45]. The staining of fractionated cells with May-Grünwald and Giemsa solutions revealed that more than 92% of the cells were neutrophilic granulocytes. The hematological and clinical findings of patients are presented in Table 1.

**Table 1:** Hematological and Clinical Findings of Patients.

No.	Age/ sex	Subtype	WBC (×10^9^/L)	Neutrophils (×10^9^/L)	Hb (g/dL)	PLT (×10^10^/L)	Cytogenetics	Therapy
1	70F	RCMD	2.1	0.8	9.2	13.4	46,XX,der(21)t(1;21)(q21+q22)	EPO
2	88F	RCMD	2.8	1.7	9.8	2.7	47,XX,+8; 46,XX	transfusion (PLT)
3	71F	RCMD	2.3	1.4	9.7	21.9	46,XX	CyA
4	79M	RCMD	1.3	0.2	7.4	14.7	46,XY	transfusion (RBC)
5	48F	RCMD	2.5	1.5	11.1	4.9	46,XX,+8	PSL
6	69F	RCMD	3.1	1.5	9.4	10.2	46,XX	(insulin, levothyroxine Na)
7	69F	RCUD	4.1	1.4	9.7	14.5	46,XX,add(13)(q12); 46,XX	transfusion (RBC, PLT), CyA
8	62M	RCUD	6.7	3.6	13.4	31.5	46,XY,del(20)(q12q13); 46,XY	transfusion (RBC, PLT)
9	78M	RCMD	3.3	0.5	6.3	37.0	46,XY	transfusion (RBC)
10	69M	RCMD	2.1	0.3	7.0	14.5	46,XY	transfusion (RBC)
11	72F	RAEB-1	4.8	2.6	9.4	37.7	46,XX	azacitidine
12	82F	RCMD	3.3	2.2	9.0	7.3	46,XX; 46,XX,del(13)(q?)	transfusion (RBC)
13	75F	RCMD	3.8	1.1	7.8	3.2	46,XX	transfusion (RBC)
14	73M	RCMD	2.4	1.9	6.1	2.6	46,XX	transfusion (RBC)
15	49F	RAEB-1	5.5	1.9	7.2	15.8	46,XX,del(5)(q?)+	transfusion (RBC)
16	80M	RCMD	2.1	0.9	8.3	11.3	46,XY	azacitidine, transfusion (RBC)
17	88F	RCMD	2.1	1.5	7.8	33.6	46,XY,del(5)(q?); 46,XY	transfusion (RBC)
18	88M	RCUD	4.0	3.1	6.6	15.4	46,XY	azacitidine, transfusion (RBC)
19	55M	RCMD	2.2	1.0	5.8	3.2	46,XY	transfusion (RBC)
20	67M	RCUD	4.7	2.5	11.4	17.7	46, XY,add(8)(q11.1), +add(8)	none
21	75M	RCMD	5.7	3.9	6.2	1.6	46,XY	transfusion (RBC, PLT)
22	61F	RCMD	2.9	1.0	7.4	1.8	46,XX,i(18)(q10)	G-CSF, CyA, ATG, transfusion (RBC, PLT),
23	87M	RCMD	3.0	1.0	5.7	39.5	46,XY; 45,X,-Y,+1,der(1;16)(q10;q10)	EPO, transfusion (RBC)

M, male; F, female; WBC, white blood cells; Hb, hemoglobin concentration; PLT, platelets; RBC, red blood cells; EPO, erythropoietin; CyA, cyclosporin A; PSL, prednisolone; G-CSF, granulocyte colony-stimulating factor; ATG, antithymocyte globulin.

### Ethics

The present study, and the process of securing written informed consent from the patients and healthy controls, were approved by the Ethics Committee of Fukushima Medical University (approval number: 1077), which is guided by local policy, national laws, and the World Medical Association Declaration of Helsinki. All study participants provided their written informed consent.

### Cells

A human promyelocytic leukemia cell line HL60 was purchased from Riken BRC Cell Bank (Tsukuba, Japan) and cultured in RPMI 1640 (Wako, Mie, Japan) supplemented with 10% (v/v) heat-inactivated fetal bovine serum (FBS) (Nichirei Biosciences, Tokyo, Japan). The cell concentration of HL60 cells and granulocytes was adjusted to 0.5 × 10^6^ and 4 × 10^6^ /mL in the medium, respectively, for incubation with or without 1 μM lipopolysaccharides (LPS) (Sigma-Aldrich, St. Louis, MO, USA).

### Transfections

Introduction of 50 nM each of human miR-34a-5p (mirVana miRNA mimic, Ambion, Life technologies, Carlsbad, CA, USA), human miR-155-5p (mirVana miRNA mimic, Ambion, Life technologies), and siRNA against *FOS* (Sense: GAAUCCGAAGGGAAAGGAAtt, antisense: UUCCUUUCCCUUCGGAUUCtc) into 2.4 × 10^6^ HL60 cells suspended in 800 μL Gene Pulser Electroporation Buffer Reagent (Bio-Rad Laboratories, Hercules, USA) was carried out by square-pulse electroporation (280 V, 12 msec) using a Gene Pulser (Bio-Rad). For the controls, corresponding amounts of control miRNA (Life Technologies) or control siRNA (Life Technologies) were introduced. After 40 hours, the cells were subjected to mRNA and protein quantification. The cell viability after electroporation was measured by trypan blue staining (control siRNA: 72.6 ± 8.7%, *FOS* siRNA: 65.7 ± 5.5%, with no significant difference).

### mRNA decay analysis

The miRNA-transduced HL60 cells were cultured at a concentration of 0.5 × 10^6^ cells in the presence of 50 μM of 5, 6-Dichlorobenzimidazole 1-β-D-ribofuranoside (DRB) (Sigma) with or without 200 μg/mL emetine (Sigma) for the indicated time.

### Total cellular RNA extraction and reverse transcription

Total cellular RNA was extracted using ISOGEN (NIPPON GENE, Toyama, Japan), and treated with DNase I (Takara Bio, Otsu, Japan). The cDNA for mRNA quantification was synthesized as described previously [45]. For miRNA quantification, RNA was subjected to polyadenylation followed by reverse transcription using a Mir-X miRNA First-strand Synthesis Kit (Clontech Laboratories, Inc., Mountain View, CA, USA).

### Real-time PCR

The quantification of mRNA was conducted as described previously [45]. Quantified transcripts of *FO*S and *TNFA* were normalized by *ACTB*. The primer sequences were *FOS* forward: 5’-GGGATAGCCTCTCTTACTACCACT-3’, *FOS* reverse: 5’-CCTCCTGTCATGGTCTTCACAAC-3’, *TNFA* forward: 5’-CCCAGGGACCTCTCTAATCA-3’, *TNFA* reverse: 5’-AGCTGCCCCTCAGCTTGAG-3’ *ACTB* forward: 5’- CAAGAGATGGCCACGGCTGCT-3’, and *ACTBn* reverse: 5’- TCCTTCTGCATCCTGTCGGCA-3’. The extraction and reverse-transcription of miRNA was conducted according to the manufacturer’s instructions (Clontech), and amplified using an miRNA-specific primer and mRQ 3’ primer (Clontech) and normalized by U6.

### Cell lysate preparation and western blotting

Cytoplasmic and nuclear lysates were prepared as previously described [24]. To prepare total cell lysates from granulocytes, the cells were precipitated in 10% trichloroacetic acid (Wako) for 30 min on ice. The TCA-precipitated fraction was treated with a lysis solution containing 9 M urea, 2% Triton X-100, and 1% dithiothreitol, and was disrupted by ultrasonication, followed by an addition of 2% lithium dodecyl sulfate and further ultrasonication. The proteins were separated on a 10% polyacrylamide gel, transferred onto Immobilon-P Transfer Membranes (Millipore, Billerica, MA, USA), and blotted with rabbit polyclonal anti-c-Fos antibody (Santa Cruz Biotechnology, Santa Cruz, CA, USA) or rabbit polyclonal anti-actin (Sigma), followed by incubation with horseradish peroxidase-conjugated anti-rabbit IgG (Santa Cruz). Signals were detected by ECL Western Blotting Detection Reagents (GE Healthcare, Buckinghamshire, UK).

### Flow cytometry

Isolated granulocytes stained with phycoerythrin-labeled anti-CD16 (Beckman Coulter, Marseille, France) or isotype IgG (Beckman Coulter) were fixed and perforated using an Intraprep Permeabilization Reagent (Beckman Coulter). After incubation with anti-c-Fos (Santa Cruz) or isotype rabbit IgG (Santa Cruz), goat anti-rabbit IgG labeled with FITC (Abcam, Cambridge, UK) was added, and the expression of c-Fos in CD16^+^ cells was analyzed by FACSCanto II (BD Biosciences, San Jose, CA, USA).

### Enzyme-linked immunosorbent assay (ELISA)

The concentration of TNF-**α** in the culture medium was measured using Quantikine HS human ELISA TNF-**α** (R&D Systems, Inc., Minneapolis, MN, USA) according to manufacturer’s instruction.

### CHIP assay

Cells were fixed with 37% formaldehyde, lysed by RIPA buffer, and sonicated. After preclearing with agarose beads, the cell lysate was incubated with 5 μg rabbit anti-human NF-κB p65 (Santa Cruz) or isotype IgG (Santa Cruz). The IgG was gathered by sperm DNA-treated agarose beads, followed by treatment with RNase A (Takara) and proteinase K (Roche Applied Science, Mannheim, Germany). The *TNFA* DNA promoter that coprecipitated with p65 was quantified by real-time PCR using a forward primer (5’-AGTCAGTGGCCCAGAAGACC-3’) and a reverse primer (5’-GGCGGGGAAAGAATCATTCAACC-3’).

### Statistical analyses

Comparison between the two groups was performed by a Mann-Whitney *U* test or paired *t* test. Data from three groups were compared using ANOVA (IBM SPSS Statistics, 17.0). *P* values less than 0.05 were considered significant. Regarding miRNA and c-Fos protein levels in individuals, the criteria for a significant increase and reduction were set as higher and lower expressions than two standard deviations from mean values of the healthy controls, respectively.

## Results

### Expression of miRNAs that possibly target *FOS* mRNA in MDS

**Fig 1:**
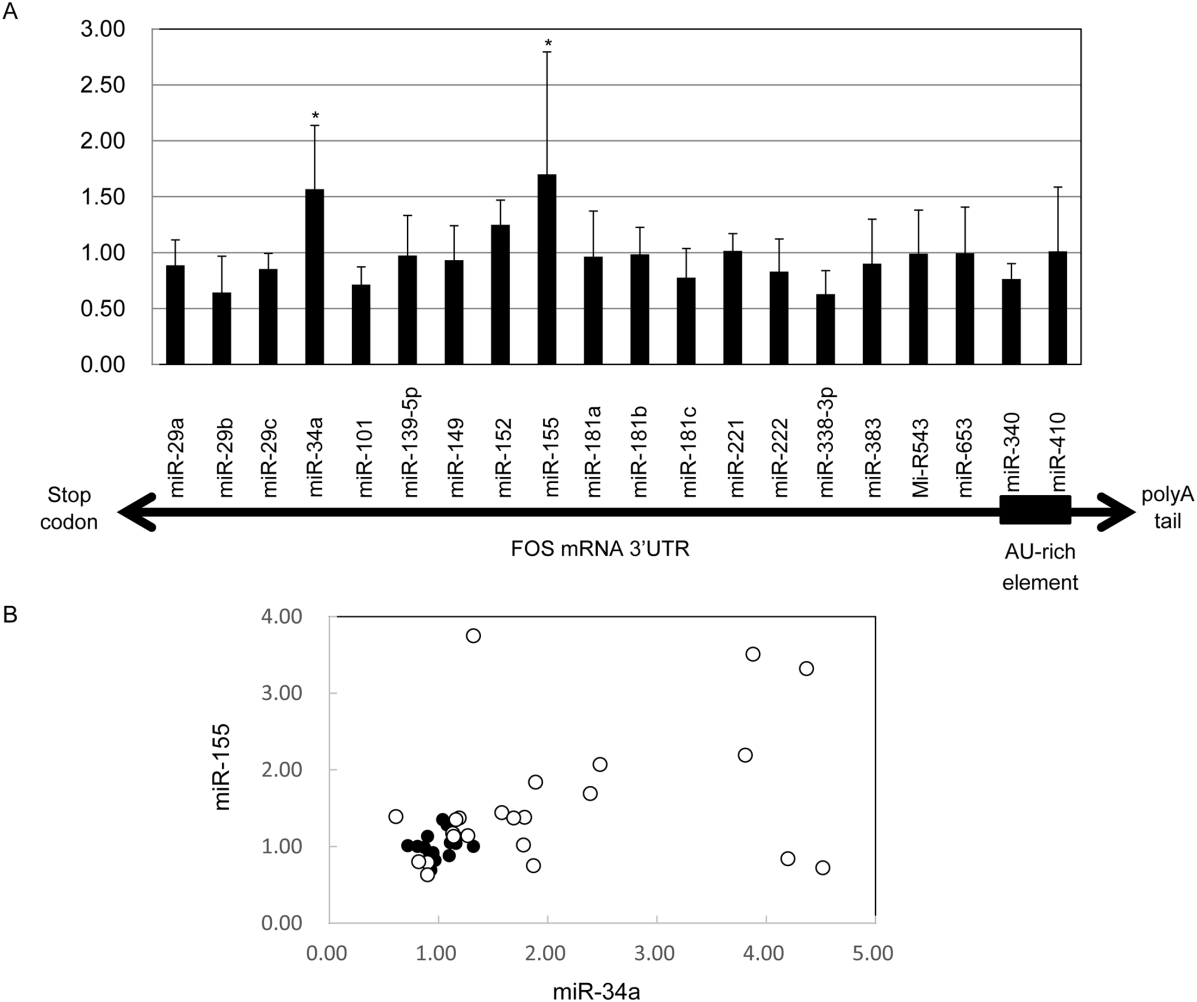
Relative expression levels of miRNAs in neutrophils. (A) Twenty miRNAs, which were predicted to bind to *FOS* mRNA 3’UTR, were quantified by real-time PCR. On the X axis, miRNAs were lined up according to the location of their binding site. The average expression levels in neutrophils isolated from six healthy volunteers were set as 1.00, and the graph presents mean values with standard deviation (SD) of six MDS patients. *; *P* < 0.05 compared with the healthy controls. (B) Expression of miR-34a and miR-155 in neutrophils from 17 healthy volunteers and 23 MDS patients. The closed and open circles represent the healthy controls and patients, respectively.

We first computationally searched for miRNAs that possibly bind to *FOS* mRNA 3’UTR, using four different databases; microRNA.org, TargetScan, Miranda, and Microcom. We found that twenty individual miRNAs were each listed by two or more databases. We quantified the expression levels of the 20 miRNAs in granulocytes derived from six patients and six healthy controls. Among them, the expression of miR-34a (1.60 ± 0.57-fold, *P* < 0.05) and miR-155 (1.70 ± 1.10-fold, *P* < 0.05) was higher in MDS neutrophils than in healthy cells (miR-34a: 1.00 ± 0.16, miR-155: 1.00 ± 0.17) (Fig 1A). Considering the heterogeneity of MDS, we expanded the measurement of miR-34a and miR-155 to 17 healthy controls and 23 patients. Compared with the controls, the expression of the two miRNAs in the 23 patients was largely diversified (Fig 1B). Twelve patients had an overexpression of miR-34a, which was defined as levels higher than the average level plus two standard deviations (SD) of the healthy controls, and ten patients had significantly increased miR-155. Both miRNAs were simultaneously increased in seven patients.

### Effects of overexpression of miR-34a and miR-155 on *FOS* mRNA stability

**Fig 2:**
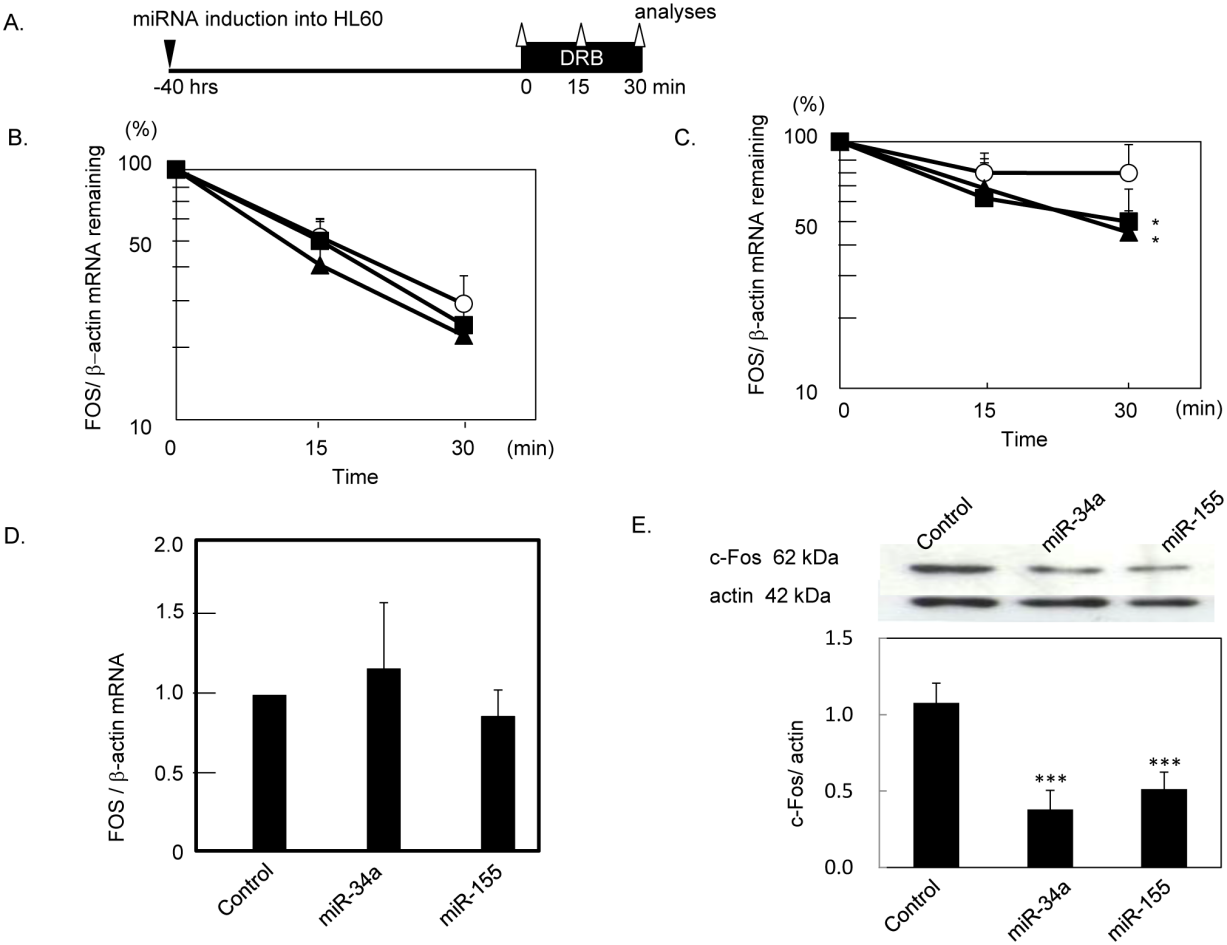
Effects of overexpressed miR-34a and miR-155 on *FOS* expression. (A) Experimental procedure. The miRNA-transduced cells were cultured with a transcription inhibitor DRB, and *FOS* mRNA was quantified at indicated time points. (B)(C) *FOS* mRNA decay in the absence (B) and presence (C) of a translation inhibitor emetine. The mean values of six independent experiments using cells which ectopically expressed miR-34a (closed squares), and miR-155 (closed triangles) or control miRNA (open circles) were plotted. Error bars indicate SD. *; *P* < 0.05. (D) Effects of miRNA overexpression on steady-state *FOS* mRNA level. Steady-state *FOS* mRNA levels were measured in miRNA-transduced cells by real-time RT-PCR. Mean values of eight experiments with SD are shown. (E) Effects of miRNA overexpression on c-Fos protein expression. The expression levels of c-Fos protein were analyzed by Western blotting and normalized by actin. The graph shows the mean values of four independent experiments. Error bars indicate SD. ***; *P* < 0.001.

To examine whether overexpression of these miRNAs caused insufficient *FOS* mRNA stabilisation under translation arrest, *FOS* mRNA decay was analysed in the presence of the transcription inhibitor DRB, using cells with exogenously introduced miR-34a, miR-155, or control miRNA (Fig 2A). *FOS* mRNA decreased to 29.0 ± 8.6% of the initial level in 30 min in the control cells, which was not altered by overexpression of miR-34a (23.9 ± 5.4%) or miR-155 (21.7 ± 6.4%) (Fig 2B). However, when translation was stopped by emetine, 74.6 ± 22.7% of *FO*S mRNA was preserved at 30 min in the control cells, while 45.3 ± 16.9% and 36.8 ± 9.5% remained in miR-34a- and miR-155-overexpressing cells, respectively, both of which were significantly lower than those in the controls (*P* < 0.05, *P* < 0.05) (Fig 2C). These results suggested that the impaired *FOS* mRNA stabilization, which we previously found in MDS granulocytes, was attributed to overexpression of miR-34a and miR-155.

### Suppression of c-Fos protein by miR-34a and miR-155 overexpression

We next attempted to experimentally examine whether miR-34a inhibited translation of *FOS*. The basal levels of *FOS* mRNA in miR-34-, and miR-155-overexpressing cells were similar to those in the control cells (Fig 2D). In contrast, exogenous expression of miR-34a or miR-155 resulted in a decreased levels of c-Fos protein by more than 50% (Fig 2E) compared with the controls, suggesting that miR-34a as well as miR-155 interfered with c-Fos translation.

### Expression of c-Fos in granulocytes of MDS patients

**Fig 3:**
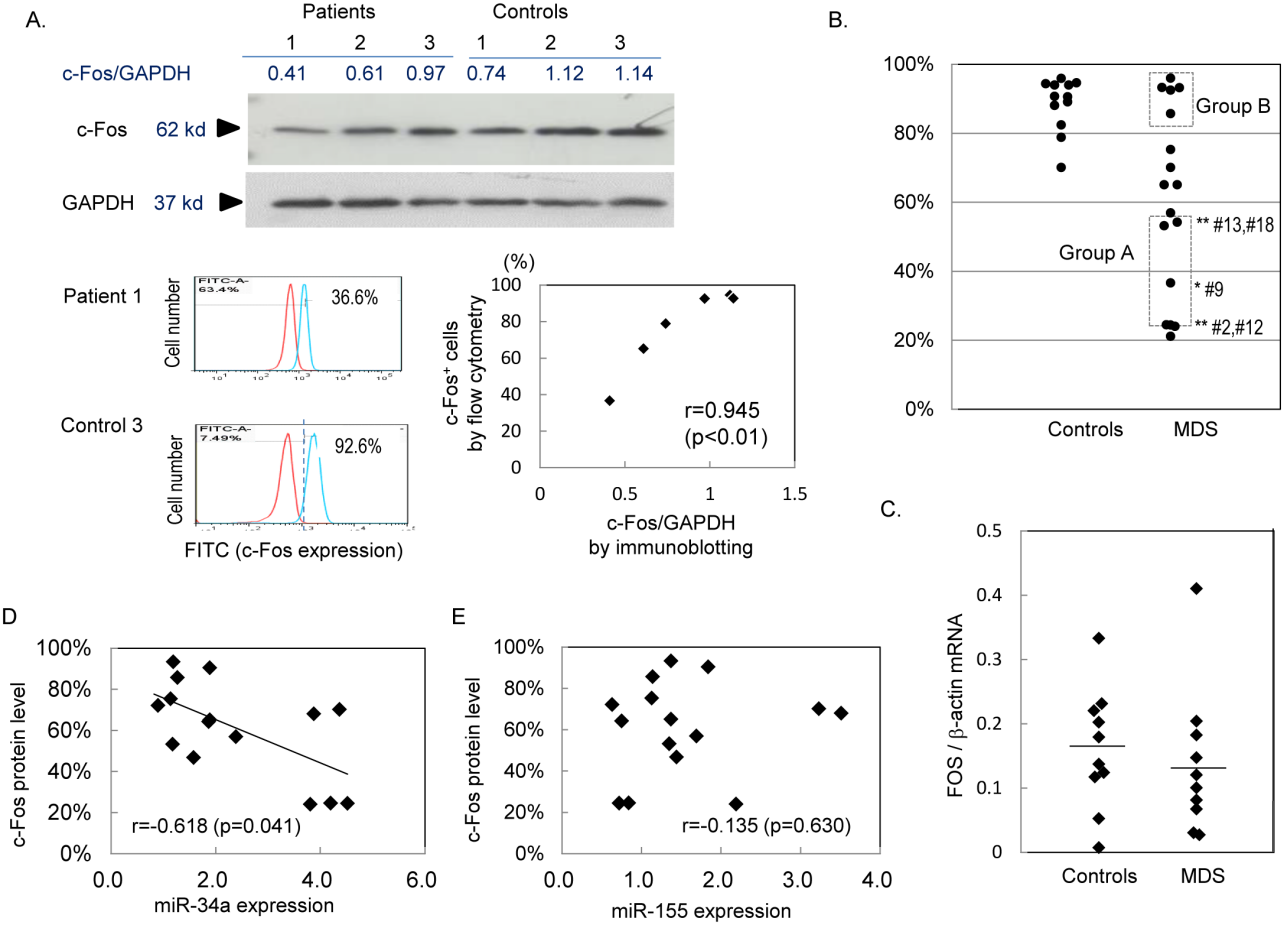
Expression of c-Fos in MDS patients. (A) Immunoblotting and flow cytometric analyses of c-Fos. Granulocytes from peripheral blood were subjected to immunoblotting. The band intensity of c-Fos was normalized by that of GAPDH, and is shown as “c-Fos/GAPDH”. For flow cytometry, granulocytes were stained with PE-labeled anti-CD16 followed by treatment with anti-c-Fos and isotype IgG labeled with FITC. The histograms obtained from Patient 1 and Control 3 are shown. The ratios of c-Fos-positive cells in CD16^+^ granulocytes were calculated, and correlation between the results from immunoblotting and flow cytometry was analyzed. (B) Comparison of ratios of c-Fos^+^ cells to total CD16^+^ neutrophilic granulocytes between the controls and MDS patients. Five out of eight patients with significantly low expressions (Group A) and another five patients with similar expression levels to those in the healthy controls (Group B) were subjected to following analyses on TNF-**α** production. The patient numbers correspond to those in Table 1. (C) Comparison of c-Fos mRNA levels. c-Fos mRNA levels in unstimulated granulocytes were compared between MDS patients and the healthy controls. (D) Correlation between c-Fos protein levels and miR-34a expression. (E) Relationship of c-Fos protein levels with miR-155.

To measure c-Fos protein levels, the neutrophilic granulocytes from MDS patients and the controls were subjected to immunoblotting and flow cytometric analyses. Since the ratios of c-Fos to GAPDH obtained by immunoblotting were well correlated with the rates of c-Fos-positive cells measured by a flow cytometer (Fig 3A), c-Fos protein levels were evaluated by flow cytometry, which requires a smaller number of cells than immunoblotting. The percentage of c-Fos-positive cells in total CD16^+^ neutrophilic granulocytes was 88.6 ± 7.8% in the healthy controls. In contrast, eight out of 17 patients tested (Patients #1, 2, 4, 7, 9, 12, 13, and 18) showed significantly low percentage of c-Fos^+^ cells, while five out of the remaining nine patients (Patients #3, 6, 8, 15, and 17) showed detectable c-Fos expression in more than 80% of cells (Fig 3B). On the other hand, *FOS* mRNA levels in granulocytes were similar in the controls and MDS patients (Fig 3C). The ratios of c-Fos^+^ in total CD16^+^ neutrophils were inversely correlated with miR-34a expression levels (r = -0.616, *P* < 0.05) (Fig 3D), but not with miR-155 levels (r = -0.135, *P* = 0.630) (Fig 3E). There were no correlation between ratios of c-Fos^+^ cells and clinical findings including white blood cell counts (r = 0.438, *P* < 0.079) and neutrophil counts (r = 0.305, *P* = 0.234). None of the medications or karyotypes were specific to the patients with significantly low c-Fos expression.

### LPS-induced TNF-α synthesis in patients with normal and low expression of c-Fos

**Fig 4:**
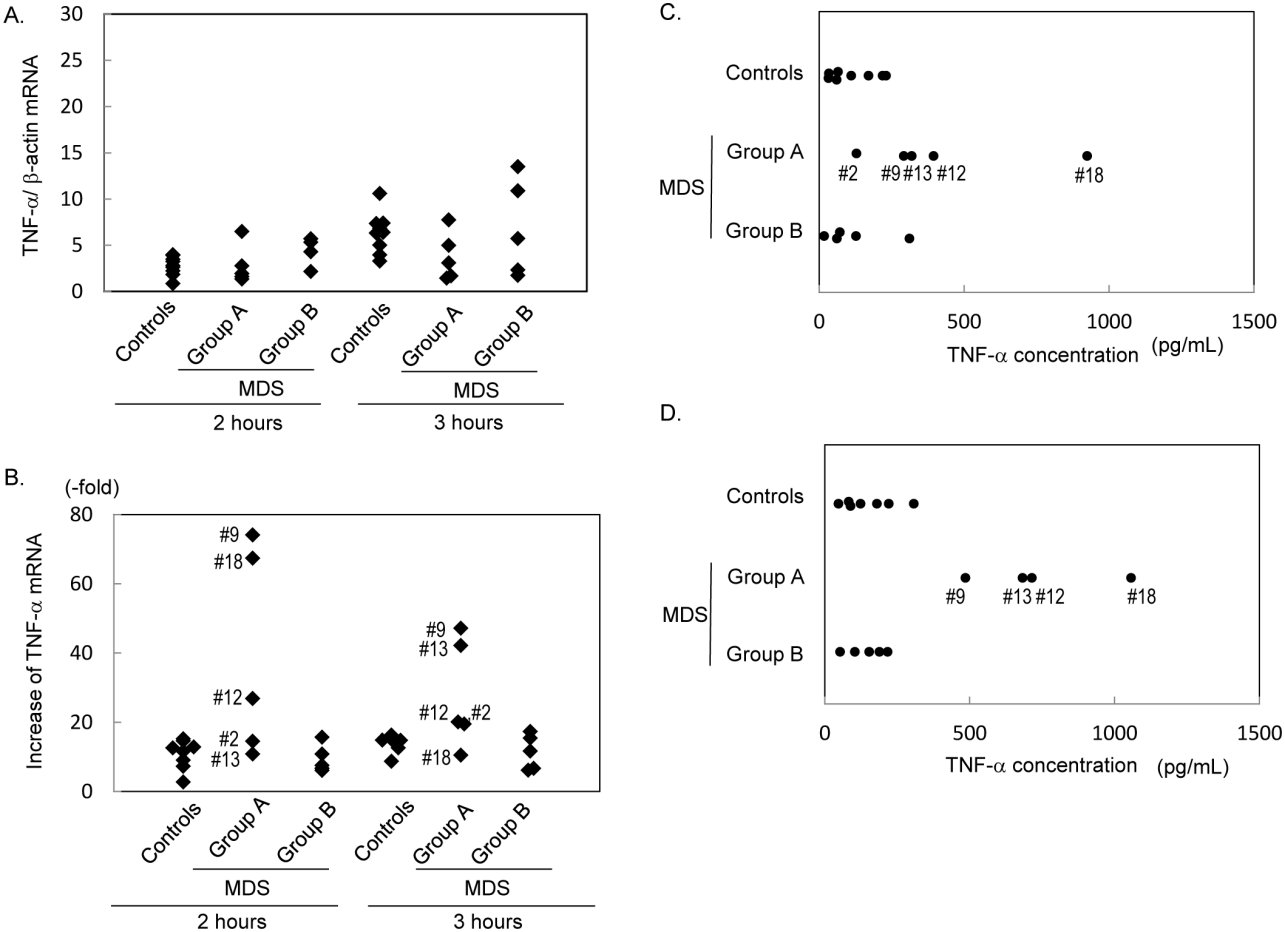
TNF-α production in response to LPS. (A) Comparison of basal levels of *TNFA* mRNA in granulocytes among healthy controls, Group A and Group B. (B) Comparison of fold increase of *TNFA* mRNA induced by simulation with 1 μg/mL LPS for indicated times. The numbers in Group A are compatible with patient numbers in Fig 3B. (C) TNF-**α** concentration in culture medium after a 2-hour stimulation with LPS. Granulocytes were cultured in the presence of 1 μg/mL LPS, and TNF-**α** concentration in medium was measured by ELISA. (D) TNF-**α** concentration in culture medium after a 3-hour stimulation with LPS.

To investigate the effects of c-Fos protein levels on TNF-**α** production in response to LPS, five patients with low c-Fos expression (Group A in Fig 3B), five with similar c-Fos levels to the controls (Group B in Fig 3B), and seven healthy controls allowed us to further draw their blood for the subsequent experiments. The miR-34a levels in the patients categorized as Group A were 2.39 (Patient #2), 1.58 (#9), 4.52 (#12), 1.89 (#13), and 1.87 (#18), when those in the healthy controls were 1.00 ± 0.15. The isolated granulocytes were then cultured in the presence and absence of LPS. Fig 4A shows the *TNFA* mRNA levels in the cells cultured without LPS for 2 and 3 hours. At both time points, no significant differences were observed in *TNFA* mRNA levels among the three groups. When stimulated with 1 μM LPS, all five patients with normal c-Fos expression in Group B showed a similar increase of *TNFA* mRNA (9.4 ± 4.0-fold at 2 hours, 11.5 ± 5.0-fold at 3 hours) to the controls (10.8 ± 4.2-fold at 2 hours, 13.9 ± 2.3-fold at 3 hours). In contrast, the patients in Group A showed a greater response than the controls at both time point (Fig 4B). The maximum increase of *TNFA* mRNA was detected at 2 hours in patients 2, 3, and 5 (26.9-fold, 74.1-fold, and 67.4-fold, respectively). At 3 hours, the increase rate of patients 1, 2, 3, and 4 ranged from 19.5 to 47.1-fold. The concentration of TNF-**α** in culture medium was also measured after 2 and 3 hours of LPS stimulation (Fig 4C and 4D). A similar TNF-**α** concentration was observed in the controls and Group B (2 hours: 115.7 ± 81.2 pg/mL in controls vs. 118.2 ± 115.4 pg/mL in Group B, 3 hours: 150.8 ± 91.5 pg/mL in controls vs. 143.5 ± 65.7 pg/mL in Group B). However, a significantly higher concentration was observed in Group A at both time points (2 hours: 412.5 ± 302.5 pg/mL; *P* < 0.05, 3 hours: 735.4 ± 237.5 pg/mL; *P* < 0.05).

### Effects of *FOS* knockdown on *TNFA* transcription in response to LPS

**Fig 5:**
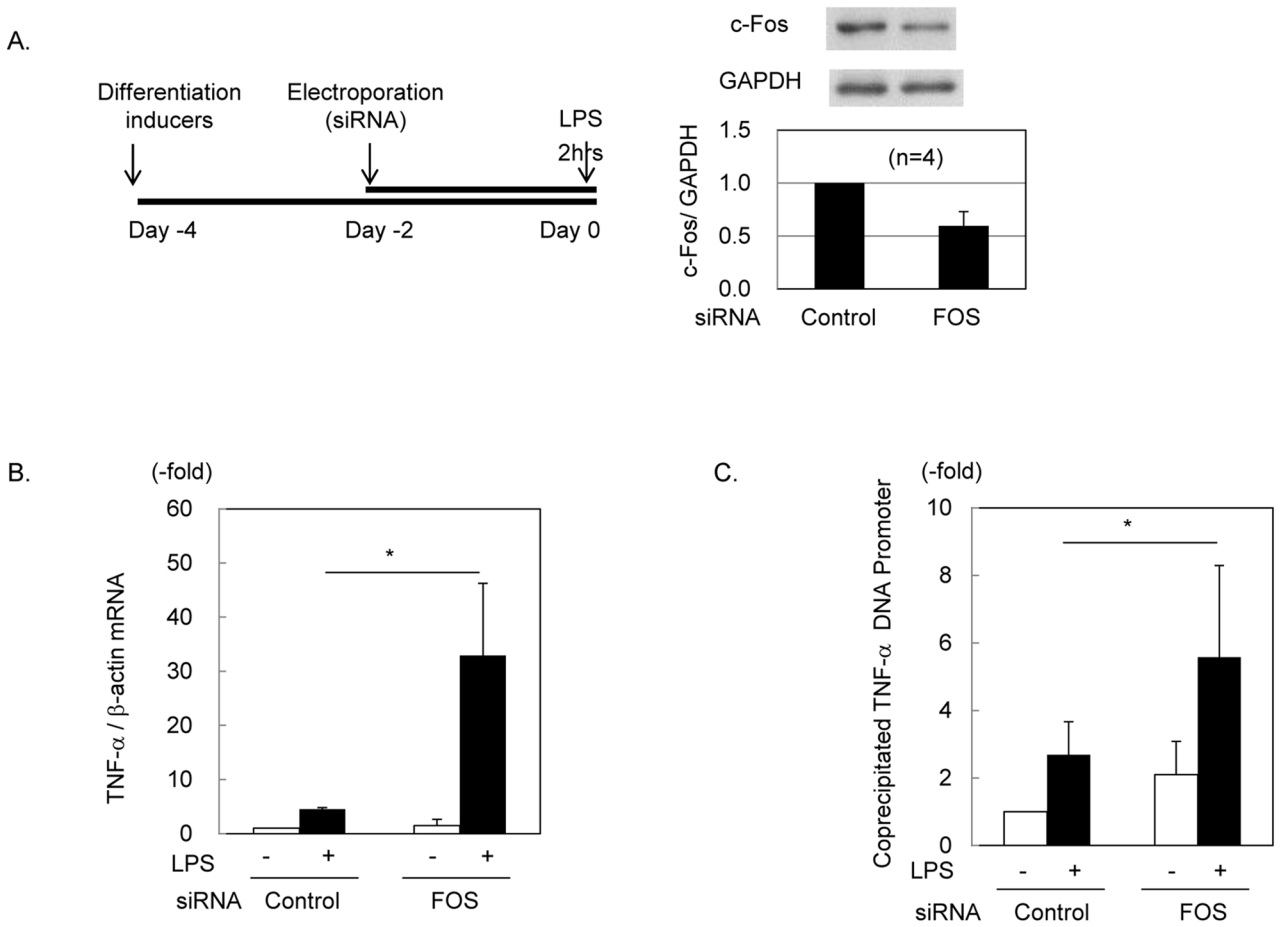
Effects of reduced c-Fos expression on TNF-α production in response to LPS. (A) Procedure of study. To induce neutrophil-like differentiation, HL60 cells were cultured with 1.25% DMSO for four days, and *FOS* siRNA or control siRNA was introduced by electroporation two days prior to the LPS stimulation. (B) Effects of c-Fos reduction on LPS-induced *TNFA* mRNA elevation. The graph shows the mean values of four independent experiments. In each experiment, the mRNA level in unstimulated control cells was set as 1.0. Error bars indicate SD. *; *P* < 0.05. (C) CHIP assay. *TNFA* DNA promoter coprecipitated with NF-κB p65 was quantified by real-time PCR. The detected amounts of promoter were compared to those from unstimulated control cells, which were set as 1.0 in each experiment. The averages of three independent experiments are shown. The error bars are SD. *; *P* < 0.05.

To confirm that reduction of c-Fos enhanced TNF-**α** production in response to LPS, c-Fos was reduced by siRNA in HL60 cells that differentiated to a neutrophilic phenotype (Fig 5A). In each experiment, c-Fos levels in *FOS* siRNA-treated cells became less than 63% of those in the control siRNA-treated cells. Without stimulation, the basal levels of *TNFA* mRNA did not differ between the control and *FOS* siRNA-treated cells. After stimulation with LPS for 2 hours, a greater increase of *TNFA* mRNA was observed in *FOS* siRNA-treated cells (32.9 ± 26.6-fold, *P* < 0.05) than in the controls (4.5 ± 2.3-fold) (Fig 5B). Since *TNFA* is transcribed by NF-κB, we examined whether c-Fos interfered with the binding of NF-κB to the promoter region of TNF-**α** DNA by CHIP assay using anti-NF-κB p65. In the *FOS* siRNA-treated cells, 2.1 ± 1.0-fold more promoter was coprecipitated with p65 in the unstimulated condition. Stimulation with LPS for 2 hours increased the binding 2.7 ± 1.0-fold in the control cells, which was significantly enhanced by reduction of c-Fos (3.6 ± 2.7-fold, *P* < 0.05) (Fig 5C).

## Discussion

To our knowledge, this is the first report to demonstrate (1) overexpression of c-Fos-targeting miR-34a and miR-155, and (2) reduced expression of c-Fos, which led to excessive TNF-**α** production in response to LPS, in MDS-derived granulocytes.

To date, aberrant expression of miR-34a and miR-155 has not been described in terminally differentiated granulocytes, although increases of miR-34a and miR-155 have previously been observed in CD34^+^ cells from patients with low- and high-risk MDS, respectively [41, 43]. Interestingly, increases of tumor-inhibiting miR-34a [38, 46] and tumor-promoting miR-155 [41, 47] were simultaneously detected.

Our data raised a new question as to what is reflected by the elevated miR-34a and miR-155 expression in granulocytes. Since miR-34a is a p53 target, the increased miR-34a in peripheral neutrophils may indicate the exposure of their progenitors to DNA-damaging stimuli that induce p53 expression. The miR-34a levels may vary according to the accumulation of damage. Some damaged progenitors may have died because of the proapoptotic feature of miR-34a [38], while some may have survived and differentiated to neutrophils. Thus, miR-34a-induced apoptosis might contribute to dispersion of miR-34a levels in MDS patients. On the other hand, it has been shown that miR-155, which is derived from a non-protein coding gene, B-cell integration cluster [48], is upregulated by NF-κB [49, 50]. Therefore, elevation of miR-155 could result from exposure to NF-κB-activating conditions, such as inflammation, oxidative stress, and endoplasmic reticulum stress. Increased expression of miR-155 is known to confer proliferative advantages by suppressing SHIP1, a negative regulator of Akt pathway [41]. To generate miR-155-high granulocytes, progenitors with elevated miR-155 need to escape from such a proliferation cycle to differentiate. The clarification as to what increased miR-34a and miR-155 could be a clue in the understanding of the mechanisms for developing aberrant granulopoiesis in MDS.

In the current study, overexpression of miR-34a and miR-155 seemed to have caused the insufficient stabilization of *FOS* mRNA in MDS granulocytes under translation arrest, which we previously reported [24]. In miR-34a- and miR-155-overexpressing cells, neither the basal level nor life span of *FOS* mRNA was altered. The mRNA destabilizing effects of these miRNAs became prominent in the presence of the translation inhibitor emetine, which suppressed endogenous mRNA degradation machinery. These phenomena were exactly the same as those which we had previously observed in MDS-derived granulocytes. In the steady state, *FOS* mRNA might be maximally degraded via multiple factors, which could mask the effects of miR-34a and miR-155.

In MDS granulocytes, elevation of miR-34a rather than miR-155 seemed to lead to reduction of c-Fos. According to the data from miR-34a-overexpressing cells, miR-34a, as well as miR-155 that had been experimentally shown to target c-Fos [25], decreased c-Fos protein level. However, an inverse correlation was observed between c-Fos protein and miR-34a but not miR-155. The lack of suppression of basal levels of FOS mRNA was common to HL60 cells that ectopically overexpressed miR-34a and MDS granulocytes. The repression of protein levels without detectable mRNA change is thought to result from incomplete complementarity between miRNA and target mRNA [51] or modest magnitude of mRNA destabilization [52].

Expression levels of c-Fos protein varied among the patients, although all samples were handled in the same way. The variety of c-Fos expression was unlikely to be due to any medications or different karyotypes, because none of them were specific to the patients with significantly low c-Fos levels. Since c-Fos levels were correlated with miR-34a, a target of p53 induced by DNA-damaging stresses, c-Fos levels may have varied with accumulation of DNA-damaging stress which the cells had been exposed to. There might be additional factors that influence c-Fos levels, because c-Fos expression is regulated post-transcriptionally at multiple steps. For example, we already reported that some MDS patients had decreased HuR levels, an mRNA binding protein that stabilizes c-Fos mRNA [24]. The decrease of HuR might affect c-Fos levels without overexpression of miR-34a. When *FOS* mRNA destabilizing proteins, such as AUF1 [35], are increased, c-Fos levels might be further decreased. Exacerbation of ubiquitin proteasome system that degrades c-Fos protein [53] could also influence c-Fos expression.

The reduction of c-Fos possibly contributes to development of ineffective hematopoiesis in both TNF-**α**-dependent and independent manners. Firstly, we confirmed that c-Fos inhibited overproduction of TNF-**α** in response to LPS in neutrophilic granulocytes, as previously shown in monocytes [15]. In HL60 differentiated to a neutrophilic phenotype by DMSO, knockdown of c-Fos enhanced the synthesis of *TNFA* mRNA, and the patients with low c-Fos expression secreted greater amounts of TNF-**α** in response to LPS than the healthy controls and the patients with normal c-Fos levels. Accumulation of excessive production of TNF-**α** under inflammatory stimuli may contribute to the formation of TNF-**α**-high condition in BM and plasma, which has been thought to induce apoptosis of hematopoietic cells in low-risk MDS [5]. Secondly, c-Fos itself is known to promote proliferation [10], and represses transcription of FasL and jun-mediated Fas [54]. Therefore, loss of c-Fos may provide hematopoietic cells with proliferative disadvantage and poor survival.

Low c-Fos expression may also affect the prognosis of patients with low-risk MDS via deregulated production of TNF-**α**. Also, c-Fos knockout mice administered LPS showed higher mortality accompanied by greater increase of inflammatory cytokines including TNF-**α** than control mice [55, 56]. Although there have been no reports that compared cytokine secretion under infection of Gram-negative bacteria between MDS patients and controls, excessive production of inflammatory cytokines may deteriorate the prognosis of MDS patients, as observed in *FOS* knockout mice exposed to LPS, which showed higher mortality accompanied by greater TNF-**α** concentration [56].

The enhanced binding of NF-κB to *TNFA* promoter by knockdown of c-Fos suggests that c-Fos interferes with NF-κB p65. This result was consistent with the previous report that showed interference of c-Fos and NF-kB p65 [15]. It is likely that cells with reduced c-Fos expression are susceptible to other NF-κB-activating stimuli, such as stresses by oxidants, irradiation, and the accumulation of misfolded proteins. Thus, not only inflammation but also various cellular stresses could be involved in the upregulation of TNF-**α**in BM and plasma via insufficient expression of c-Fos.

## Conclusions

In the present study, we demonstrated the reduction of c-Fos via overexpression of miR-34a in MDS granulocytes, which to our knowledge has not been reported in hematopoietic diseases before. The low levels of c-Fos resulted in excessive production of TNF-**α**, which is considered to be a contributing factor to the development of ineffective hematopoiesis. Further studies on the mechanisms of aberrant miRNA upregulation and TNF-**α** overproduction would provide insights to unveil the pathophysiology behind ineffective hematopoiesis in MDS.
